# A Rare Localized Pituitary Stalk Germinoma Presenting in the Third Decade

**DOI:** 10.1155/2018/1746917

**Published:** 2018-12-17

**Authors:** Alex González Bóssolo, Michelle Mangual Garcia, Kyrmarie Davila, Ricardo Brau, Javier Sanchez Ortiz, Jose Hernan Martinez

**Affiliations:** ^1^Section of Medicine, Endocrinology, Department of Diabetes and Metabolism, San Juan City Hospital, San Juan, Puerto Rico, USA; ^2^Section of Neurosurgery, Department of Surgery, School of Medicine, University of Puerto Rico Medical Sciences Campus, San Juan, Puerto Rico, USA; ^3^Pathology Department, University of Puerto Rico School of Medicine, San Juan, Puerto Rico, USA

## Abstract

We report the case of a 34-year-old male Hispanic patient who presented with an 8-month history of polyuria and decreased libido. An evaluation revealed hypopituitarism, central diabetes insipidus, and a pituitary stalk lesion. No evidence of a neoplasm or an inflammatory/infiltrative disease was found. We treated the patient conservatively with steroid therapy. However, as a result of disease progression, transsphenoidal surgery was performed to obtain tissue for a pathological analysis. Histopathology revealed an intrasellar germinoma, confirmed by immunochemistry. Due to localized disease, radiotherapy was initiated and clinical improvement was noted. This case emphasizes the importance of histopathological analysis; for many physicians, the presentation of a pituitary stalk lesion in a young male adult creates a clinical conundrum. Although the most common etiologies are an inflammatory or secondary neoplasm, the possible presence of an intrasellar neoplasm should not be ruled out. In many cases, imaging characteristics and tumor markers may aid in the diagnosis without the need of an aggressive diagnostic approach. However, in this case, histopathological analysis was the only way to make a correct diagnosis and provide adequate treatment for the patient.

## 1. Introduction

Germ cell tumors are common neoplasms that occur within the pediatric population. A failure in cell migration to the genital crest can lead to an extragonadal presentation [1]. While the most common central nervous system (CNS) site for germ cell tumor growth is the pineal gland [1], growth in a patient's third decade of life and an intrasellar presentation are both unconventional features [2]. We present the unique case of a 34-year-old man with pituitary stalk inflammation, secondary to a germ cell tumor.

## 2. Case Presentation

A 34-year-old man without a previous medical history was referred to our endocrinology clinic due to an eight-month history of generalized fatigue, increased thirst, increased urinary frequency and volume, reduced nocturnal erections, and decreased sexual desire. Additionally, he reported a weight loss of 18 kg in six months, dry skin, cold intolerance, and constipation during the same period. He did not report any headaches, vision problems, recent head trauma, use of illegal substances, family history of pituitary diseases, or close contact with anyone sick. At the time of his referral, therapy with cortisone acetate 25 mg twice daily and levothyroxine (LT4) 50 mcg daily had already been initiated by his primary care physician.

At the initial visit, physical examination revealed normal vital signs and a delayed tendon reflex of the deep tendon reflex in the upper/lower extremities. The first set of serum and urine laboratory examination results revealed hypopituitarism, hypogonadotropic hypogonadism, polyuria, and hypertonic hypernatremia. The first magnetic resonance imaging (MRI) scan showed a right-sided pituitary microadenoma (3 mm). No suprasellar mass or compression of the optic chiasm was noted. Steroid therapy was changed to hydrocortisone 10 mg/5 gm and levothyroxine replacement was optimized by weight. Follow-up serum laboratory and imaging studies were ordered.

**Figure 1:**
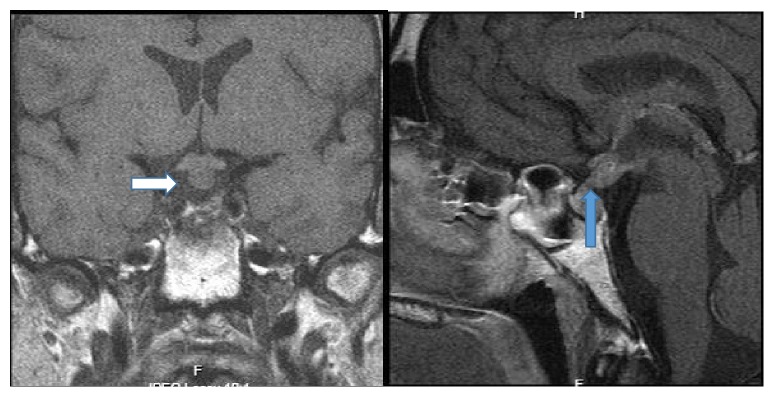
Magnetic resonance imaging (MRI) with gadolinium.** A:** T1-weighted showing diffuse thickening of the infundibulum (white arrow).** B:** T1-weighted sagittal showing absence of the posterior pituitary bright spot (blue arrow).

A second MRI, one month later, revealed an abnormal thickening of the infundibulum with no evidence of the microadenoma (Figure 1). New laboratory examination results confirmed the previous findings and ruled out the presence of an autoimmune or inflammatory condition (Table 1). Tumor markers for germ cell tumors were negative. A water deprivation test confirmed central diabetes insipidus. A chest X-ray was unremarkable for masses or infiltrative lesions. Testosterone replacement and desmopressin (DDAVP) were added.

**Figure 2:**
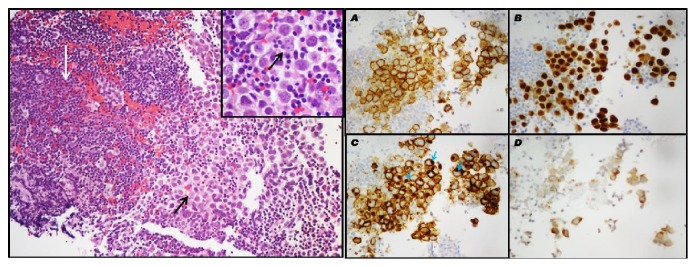
Germinoma (left): large round germ cells (black arrows) within a background of lymphocytes (white arrow); H&E 200x, insert showing H&E 400x (right).** A:** C-kit (CD117) stains the membranes of neoplastic cells (400x).** B: **OCT-4 producing strong nuclear staining (400x).** C: **PLAP staining the membranes and the Golgi apparatus, blue arrows (400x).** D: **B-hCG staining isolated syncytiotrophoblastic cells (400x). B-hCG: human chorionic gonadotropin.

Due to nonspecific imaging findings, a negative X-ray, and no evidence of systemic disease, a conservative approach with prednisone was taken. However, six months later, the stalk lesion worsened. At that time, the patient underwent transsphenoidal surgery for a tissue biopsy. The tissue was positive for an intrasellar pituitary germinoma, confirmed by positive human chorionic gonadotropin (hCG) staining, C-kit, and placental alkaline phosphatase (PLAP) (Figure 2). A lumbar MRI scan and a lumbar puncture were negative for metastatic disease. Treatment with whole ventricle brain radiation was started, with subsequent clinical improvement.

## 3. Discussion

Primary intracranial germ cell tumors are neoplasms that predominantly occur within the pediatric population and account for 0-4-3.4% of primary intracranial tumors [7]. A failure in cell migration to the genital crest can lead to an extragonadal origin [1]. However, the majority of cases occur in the pineal or suprasellar compartment, with a peak incidence between ages 10-21 [8]. The literature on parasellar germinomas in patients over age 30 is scarce and primarily consists of case reports [1, 9]. In literature of patients over the age of 30, males seem to be more affected than females, a finding which aligns with the SEER registry of intracranial germinomas in patients aging 0-29 years old [10]. According to meta-analyses conducted on treatment guidance of intracranial germinomas, this isolated germinoma presentation in a male over 30 years old is practically nonexistent [11–14].

The presentation described in our case creates a clinical challenge for physicians. Our initial concern for this patient was the reported decreased libido and polyuria. These symptoms are common in patients with stalk lesions. In a retrospective cohort, Young et al. described 152 patients with pituitary stalk lesions; in this cohort, hypogonadism was the most common endocrine abnormality, followed by diabetes insipidus [15]. However, clarifying the etiology of the lesion for proper diagnosis and treatment is burdensome. This mainly occurs due to the imaging findings of stalk thickening. This finding arose due to a lymphohistiocytic inflammation in the cerebrospinal fluid that caused pituitary stalk inflammation [16]. Although some stalk lesion presentations and features might favor some etiologies, most of them are nonspecific; this creates a clinical conundrum [15]. Also other etiologies such as Langerhans cell histiocytosis [LCH] are indiscernible from a germinoma by MRI [17, 18]. Therefore, as in our case initially, the majority of reported cases are treated as lymphocytic hypophysitis [3, 4] (Table 2). Secreted tumor markers, such as alpha-feto protein and hCG, can aid in the diagnosis. Unfortunately, these markers are not always present in the serum or cerebrospinal fluid (CSF) [19]. Our patient's results were consistent with the study by Seregni et al., in which 33% of the patients with CNS germinoma presented with normal hCG and alpha-feto protein levels in serum and CSF [20].

Our clinical judgment was consistent with the report by Young et al. As the imaging findings were nonspecific, there was no evidence of systematic disease, and normal tumor markers were present, a conservative approach was taken, and the patient was treated with glucocorticoids. However, when progression of the disease with a further enlargement of stalk lesion on MRI was noted six months later, an aggressive procedure was required and subsequently confirmed the diagnosis. Following diagnosis, we were able to administer adequate therapy. As our patient had a localized germinoma, radiotherapy (RT) was highly effective. Studies have shown a 5-year progression-free survival (PFS) rate of more from 88-97% [20]. Although adjuvant chemotherapy has been used to minimize radiotherapy effects, the PFS has lessened in cases of using focal RT rather than whole brain RT [21]. Additionally, the efficacy of chemotherapy alone has been shown to be inferior to that of radiotherapy, with a 44% rate of recurrence at 2.5 years after therapy [22].

In conclusion, the presentation of a pituitary stalk lesion in a young male adult creates a clinical challenge for most physicians. Although the most common presentation is an inflammatory or metastasis, the presence of intrasellar pathology should not be discarded. A conservative approach should be pursued first, reserving a more aggressive approach for cases with equivocal imaging and biochemical findings. However, in cases similar to ours, a histopathological analysis may be the only way to determine a correct diagnosis and provide adequate treatment for the patient.

## Figures and Tables

**Table 1:** Laboratory values.

Laboratory Test	Results
Ferritin (ng/mL)	242.80
Thyroid Stimulating Hormone (*μ*IU/mL)	1.44
Free T4 (ng/dL)	0.9
Adrenocorticotropic Hormone (pg/mL)	15
Cortisol AM (*μ*g/dL)	2.38
Total Testosterone (ng/dL)	< 0.10
Urine Volume (mL/24 hours)	11225
Urine Creatinine (mg/24 hours)	1582.7
ANA test	Negative
HIV 1/2 Antigen Antibody Fourth Generation	Nonreactive
Prolactin (ng/mL)	12.1
Anti-TPO Microsomal (IU/mL)	6.84
Thyroglobulin AB (IU/mL)	11.01
Iron - Total Serum *μ*g/dL	93
% Sat of Iron	29
Ferritin (ng/mL)	242.80
AST (U/L)	19
ALT (U/L)	9
Alpha-Fetoprotein Tumor Marker (ng/dL)	< 1.3
B-HCG (mIU/mL)	< 0.05
Immunoglobulin G Subclass 4 (mg/dL)	21.3
Proteinase 3 Antibody	< 1.0
Myeloperoxidase Antibody	< 1.0

B-HCG: human chorionic gonadotropin, AST: aspartate transaminase, ALT: alanine transaminase, TPO: thyroid peroxidase, and AB: antibody.

**Table 2:** Cases of germinomas with similar clinical features described in the literature.

**Case**	**Age**	**Sex**	**Clinical Feature**	**Hormonal Deficiency**	**Stalk Thickening MRI**	**Treated as LH**	**Tumors Markers**
Terasaka et al. (2012) [3]	40	Female	Headache, diplopia	Hypopituitarism, Central DI	Yes	Yes	PLAP
Torremocha et al. (2002) [4]	45	Male	Headache	Gonadotropin deficiencies	Yes	Yes	b-hCG [CSF]
Shimizu et al. (2014) [5]	36	Male	General malaise, decreased libido	Hypopituitarism, Central DI	Yes	No	None
Wen-Ping Yang et al. (2017) [6]	38	Male	General weakness	Hypopituitarism,	No	No	b-hCG [CSF]
Gonzalez et al. (2018)	34	Male	Fatigue, decreased libido	Hypopituitarism, Central DI	Yes	Yes	PLAP, C-kit, b-hCG

PLAP: placental alkaline phosphatase, DI: diabetes insipidus, b-hCG: beta-human chorionic gonadotropin, CSF: cerebrospinal fluid, MRI: magnetic resonance imaging, and LH: lymphocytic hypophysitis.
